# Oral Anticoagulants and Renal Impairment: The Convoluting Dilemma

**DOI:** 10.1016/j.ebiom.2016.04.027

**Published:** 2016-04-27

**Authors:** Victor Serebruany

**Affiliations:** Department of Neurology, Johns Hopkins University, Osler Medical Building, 7600 Osler Drive, Suite 307, Towson, MD 21204; USA

The optimal utilization of oral anticoagulants (OAC) in patients with renal impairment (RI) represents an urgent, unmet, and yet unsolved need with regard to the choice of agents, duration of treatment, and potential dose/regimen adjustment (De Caterina et al., 2016, Verheugt and Granger, 2015). Lack of any large randomized trials adequately designed and powered specifically in such high-risk patients, absence of the uniformed efficacy and safety data reporting policy to the government agencies, and endless overoptimistic publications based on post hoc analyses of primary mega trials sometimes exaggerating benefits and hiding risks cloud the reality. In addition, triaging RI patients are problematic due to ongoing kidney deterioration, and the fact that such patients are simultaneously prone to both vascular occlusions and bleeding (Blann & Lip, 2015). Despite significant reductions in morbidity and mortality over the last half-century, residual vascular risk remains disproportionately high in the RI population. Our inability to assess adequately the impact of OA on long-term outcomes in these patients has been well recognized e.g. (De Caterina et al., 2016, Verheugt and Granger, 2015, Blann and Lip, 2015, Ng et al., 2013).

Most of the evidence triaging OAC in RI patients consists of subgroup analyses of trials, which in turn usually exclude patients with severe RI. Not only are the sample sizes of most RI subgroups in such trials are woefully small, but also the definitions of RI are variable making cross-trial comparisons and definite conclusions difficult. In addition, many OAC trials deliberately avoided enrolling RI patients, especially those with end stage renal failure or requiring dialysis or/and kidney transplantation. To make the story even more complicated, RI patients are prone to both thrombotic vascular occlusions and excess bleeding makes the task of finding an optimal OAC regimen a variation of “mission impossible” for such high-risk population. Regarding the impact of OAC on efficacy, the scant evidence suggests some positive impact on a reduction of stroke but with uncertain effects on mortality and consistently increased bleeding rates. Furthermore, definitions of events are constantly changing especially with respect to bleeding, with rates varying greatly depending upon the scales/classifications used. In addition, trial durations and evolving standards of care are heterogeneous making the historic comparisons even more challenging. Most experts agree that the benefits of OAC in RI are uncertain and may be potentially outweighed by bleeding hazards, while acknowledging several serious gaps in evidence. Hence risks may outweigh benefits among people with low annual rates of stroke including those with early stages of RI, especially in patients who do not have clinically-evident vascular disease. Managing OA in such high-risk population is tricky since RI patients experience increased hemostatic activation but attenuated response to OA compared with patients without RI, even despite higher dosages (Ng et al., 2013, Shen et al., 2012).

In this issue of *EBioMedicine*, Proietti and colleagues (Proietti et al., 2016) analyzed pooled datasets (n = 3646) from SPORTIF III and V trials of warfarin-treated patients dependent on renal function. Diminished creatinine clearance (< 60 ml/min) was reported in 952 (26%) patients. Overall, the time in therapeutic range (TTR) was higher in patients with normal renal function compared to those with RI (p < 0.001). By logistic regression, chronic atrial fibrillation and male gender were associated with TTR > 70%, whilst diabetes mellitus, aspirin use and RI were inversely associated with TTR > 70%. On Cox analysis, RI was an independent predictor for stroke (p = 0.006) and death (p < 0.001); while TTR > 70% was independently associated with a lower risk of stroke (p = 0.024), death (p = 0.001) and major bleeding (p = 0.001). The combined SPORTIF warfarin data suggest that RI is highly prevalent among patients with atrial fibrillation, being a risk factor for stroke and death. Adjusting for RI, good quality anticoagulation control (TTR > 70%) was an independent predictor for lower risks of stroke, death and major bleeding.

There are few important considerations yielded from these latest elegant data. Importantly, patients with even mild RI experience much more frequent vascular thrombotic events than patients with normal kidney function. The index data are in full agreement that despite huge differences among the trials with regard to exclusions, baseline characteristics, randomization or enrollment patterns, and length of follow up, RI patients have consistently higher risks to experience primary vascular endpoint event despite even a very liberal eGRF or creatinine clearance of < 60 ml/min cut-off to triage RI cohorts. Also, the RI patients experience much more frequent bleeding events, especially those with non-adjusted TTR. These two disturbing findings raise obvious concerns that we may consider OAC dose/regimen downgrades in such patients, the strategy which is currently not recommended by the regulatory agencies. The problem is that RI patients constitute no > 10–15% of the entire trial pool generating woefully small dataset(s) for each particular OAC. Dichotomizing patients further into severe, mild, or moderate RI, make such groups very small, usually in double-digit numbers hence preventing quality analyses. These circumstances allow the regulatory authorities to ignore such obvious shortcomings, or/and demand unbiased risk assessment in RI patients receiving OAC. Indeed, the fact that patients exhibited such poor outcomes in RI, (Proietti et al., 2016) we should consider the advantage of non-Vitamin K antagonist oral anticoagulants (NOACs) over warfarin. Indeed, there is some evidence (NICE Guidance, n.d, Halvorsen et al., 2014) that NOACs may be superior to warfarin causing less bleeding complications. However, all OAC mega trials suffer from massive double-digit incomplete follow-up rates, challenging the quality of the analyzed datasets (Marciniak et al., 2016). Overall, the current knowledge suggests no single superior OAC choice with regard to their safety and efficacy in patients with RI (Harel et al., 2015). Further comparative randomized studies of different OACs in patients with moderate and severe RI are urgently needed.

## Disclosure

The author declared no conflicts of interest.
